# Desmoglein-2 as a cancer modulator: friend or foe?

**DOI:** 10.3389/fonc.2023.1327478

**Published:** 2023-12-22

**Authors:** Kay K. Myo Min, Charlie B. Ffrench, Barbara J. McClure, Michael Ortiz, Emma L. Dorward, Michael S. Samuel, Lisa M. Ebert, Mỹ G. Mahoney, Claudine S. Bonder

**Affiliations:** ^1^ Centre for Cancer Biology, SA Pathology and the University of South Australia, Adelaide, SA, Australia; ^2^ Adelaide Medical School, University of Adelaide, Adelaide, SA, Australia; ^3^ Basil Hetzel Institute, Queen Elizabeth Hospital, SA, Adelaide, Australia; ^4^ Royal Adelaide Hospital, Adelaide, SA, Australia; ^5^ Department of Pharmacology, Physiology, and Cancer Biology, Thomas Jefferson University, Philadelphia, PA, United States

**Keywords:** desmoglein-2 (DSG2), cancer, cadherin, intercellular junctions, prognostic biomarker, vasculogenic mimicry, non-canonical role

## Abstract

Desmoglein-2 (DSG2) is a calcium-binding single pass transmembrane glycoprotein and a member of the large cadherin family. Until recently, DSG2 was thought to only function as a cell adhesion protein embedded within desmosome junctions designed to enable cells to better tolerate mechanical stress. However, additional roles for DSG2 outside of desmosomes are continuing to emerge, particularly in cancer. Herein, we review the current literature on DSG2 in cancer and detail its impact on biological functions such as cell adhesion, proliferation, migration, invasion, intracellular signaling, extracellular vesicle release and vasculogenic mimicry. An increased understanding of the diverse repertoire of the biological functions of DSG2 holds promise to exploit this cell surface protein as a potential prognostic biomarker and/or target for better patient outcomes. This review explores the canonical and non-canonical functions of DSG2, as well as the context-dependent impacts of DSG2 in the realm of cancer.

## 1 Introduction

Adhesion between individual cells is crucial for structural integrity of tissues, and can regulate the formation and maintenance of a tumor (1). Desmosomes are specialized junctional complexes that can provide cell-to-cell adhesion to maintain tumor mass integrity. A loss of adhesion between cancer cells within a tumor contributes to cancer invasion, dissemination, and metastasis (2). Desmogleins (DSG) and desmocollins (DSC) are the two members of the cadherin superfamily that contribute to desmosomal junctions. Within desmosomes, there are four known isoforms of desmogleins (DSG1-4) and three known isoforms of desmocollins (DSC1-3). Desmoglein-2 (DSG2) is a calcium-binding single pass transmembrane glycoprotein of the cadherin protein family. DSG2 molecules on neighboring cells can dimerize via their extracellular domains (3–5). Within the adhesive unit of desmosomes, preferential heterophilic interactions occur between desmogleins and desmocollins (e.g. DSG2 and DSC2), but homophilic DSG2 interactions have also been documented (6).

DSG2 expression has been observed in several tissue types such as the epidermis, the intestinal mucosa and myocardial cells of the heart (4). Mutations in the *DSG2* gene results in a loss of desmosomal adhesion, which has been associated with several autoimmune, infectious and hereditary disease states (7, 8).

There is increasing evidence from us and others that DSG2 regulates many cancer cell functions, including cell adhesion (9, 10), proliferation (11), migration, invasion (12), vasculogenic mimicry (13) and tumor growth (14–16). However, the precise role/s of DSG2 in cancer progression varies across different cancer types. In this article, we review the reported roles of DSG2 in cancer and detail the mechanisms by which DSG2 influences neoplastic behavior.

## 2 Tissue structure and adhesion

**Figure 1:**
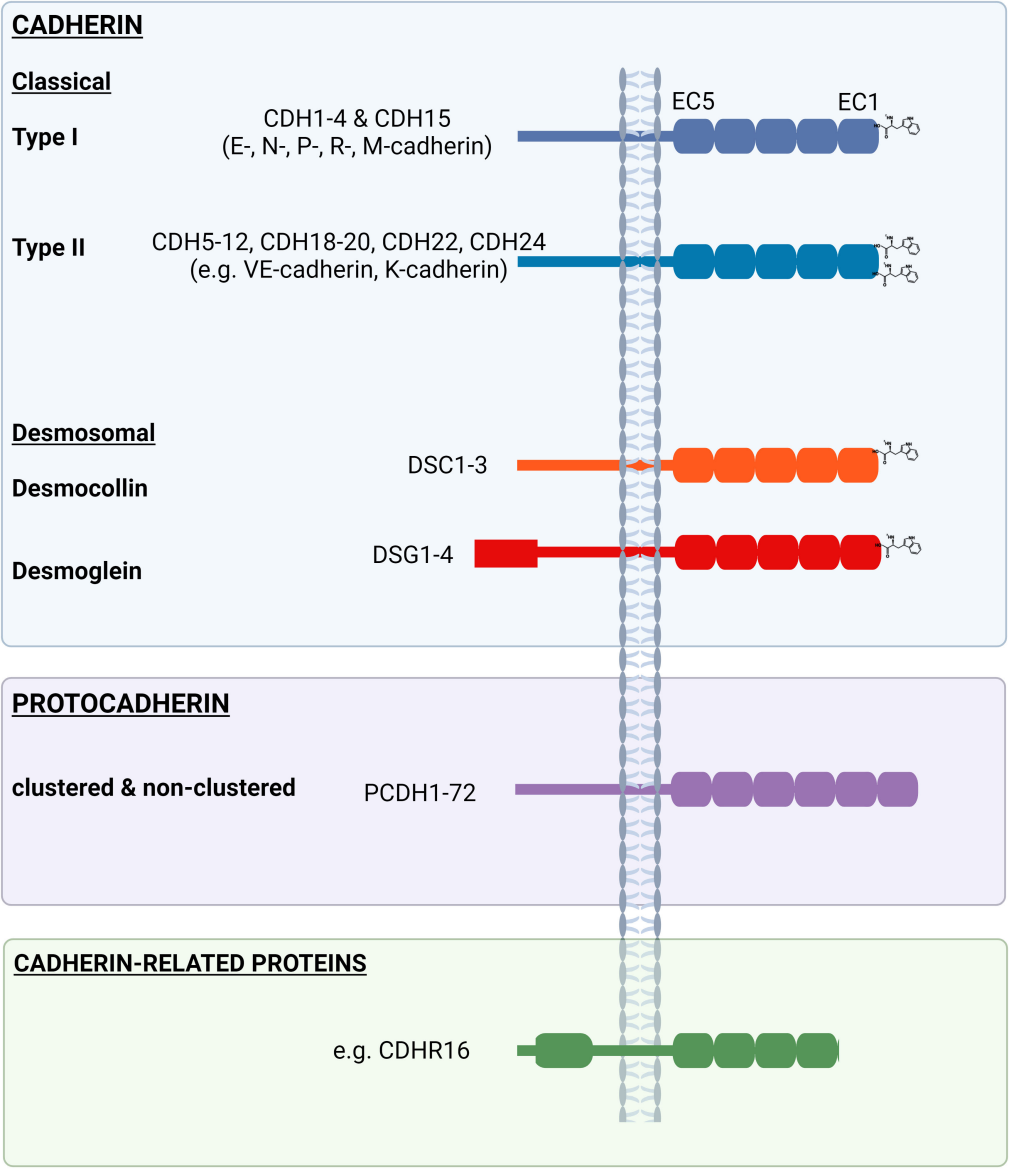
Examples of the ‘cadherin’ family comprising the ‘classical’ (type I and type II) and ‘desmosomal’ (desmogleins and desmocollins) (blue box), as well as ‘protocadherins’ (purple box’) and other ‘cadherin-related proteins’ (green box). Extracellular (EC) domains differ between and across cadherin family members, as do the length of intracellular domains.

Research into the adhesive properties of cells began almost half a century ago when Takeichi documented a calcium(Ca^2+^)-dependent process that facilitated the formation of cell-cell contacts (17). Soon thereafter, the adhesive protein ‘cadherin’ was identified at the joining edge of adjacent epithelial cells, which served to link these junctions to the actin cytoskeleton (18, 19). The discovery of over 100 cadherin-like proteins revealed a superfamily of adhesion molecules (**Figure 1**) that collectively contribute to cell-cell recognition, intercellular adhesion and signaling pathways that are important for morphogenesis and tissue behavior.

Cadherins, such as DSG2, are cell surface proteins with an ectodomain comprised of cadherin repeats, followed by a single pass transmembrane domain and then a relatively short cytoplasmic domain. More specifically, cadherins contain at least two extracellular cadherin (EC) repeats, with each repeat comprised of ~110 amino acids and encoding an immunoglobulin-like fold of seven beta strands that form two beta sheets. The individual EC domains are largely conserved across the cadherin family and are interconnected via highly conserved Ca^2+^ binding regions. This strand of repeat units are held within the plasma membrane via a single pass transmembrane domain. Notably, the EC domain most proximal to the transmembrane region (i.e., EC5) and the intracellular region are poorly conserved across family members, with exception for the internal catenin-binding motifs. The cadherin superfamily is divided into three major groups; ‘cadherin’, ‘protocadherin’ and ‘cadherin-related proteins’ based on variations in amino acid sequence, structural properties and protein function (reviewed in (20)).

The cadherin family is further subdivided into two main groups, ‘classical’ (type I and type II) and ‘desmosomal’ (DSG and DSCs) (**Figure 1**). The classical type I (e.g., E-cadherin) cadherins, desmogleins and desmocollins all contain five consecutive EC repeats in their ectodomain and share a highly conserved tryptophan (Trp, W) at position 2 (Trp2) in EC1, which is used during adhesion by strand swapping for either cis- or trans- interactions (6). In contrast, type II cadherins (e.g., VE-cadherin) contain an additional Trp at position 4 (Trp4) in EC1 that orchestrates a homophilic interaction between partner proteins (21).

Compared to the other cadherins, the DSG cytoplasmic domain is considerably longer with an additional C-terminal DSG-specific cytoplasmic region containing an intracellular proline rich linker domain, a variable repeat unit domain (5 repeats in DSG1, 6 in DSG2, 2 in DSG3 and 3 in DSG4) and a DSG terminal domain (with the exception of DSG3, which lacks a terminal domain) (22).

**Figure 2:**
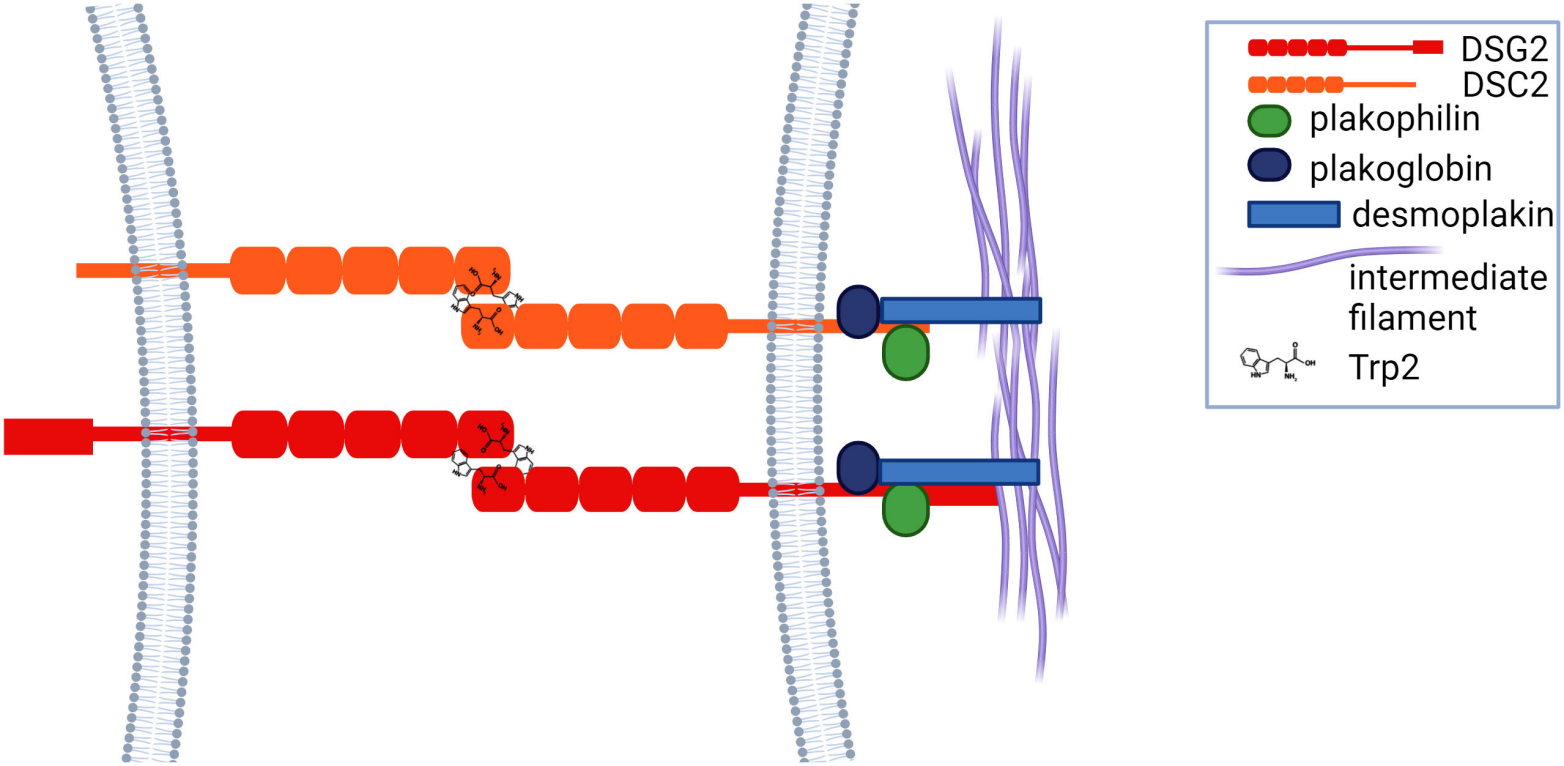
Cadherins adhere via tryptophan residues (Trp2) and strand exchange between the EC1 domains of opposed proteins, after which they engage with plakoglobin, plakophilin and desmoplakin, which is linked to intermediate filaments.

The interdomains between the EC regions are stabilized by conserved Ca^2+^ binding sites. Three Ca^2+^ ions within each interface help to maintain the EC domain in an extended rigid configuration to enable adhesive binding (23). Adhesion between cadherin proteins involves strand exchange between the EC1 domains of opposed molecules, mediated by insertion of the hydrophobic side chain of conserved tryptophan residues (Trp2) into hydrophobic pockets on the opposing molecules (22) (**Figure 2**). Intracellular domains of desmosomal proteins engage with intracellular plakoglobin, plakophilin and desmoplakin, which associates with intermediate filaments, linking the complex to the cytoskeleton, thus forming a ‘hyper adhesive’ desmosome plaque that supports cellular mechanical stress (**Figure 2**).

Desmosomal proteins, like other cadherins, can exhibit both homophilic and heterophilic adhesion. Live imaging of fluorescently tagged DSG2 demonstrated its localization to vesicles that, once mobilized to the membrane (24), are stabilized at the membrane via dimerization with plakoglobin proteins (and others) in the cytoplasmic domain (25).

## 3 Non-canonical cellular functions

Of particular focus in this review, DSG2 has demonstrated capability far beyond simply binding cells together through desmosomes. Phenotypic features identified in *Dsg2* knockout mice, including alterations in cellular differentiation and proliferation (26), are not readily attributable to DSG2’s role in cell-cell adhesion alone. Most notable is the demonstration by Eshkind et al., that complete genetic ablation of *Dsg2* in mice is embryonically lethal in the 129/Sv strain background, but not C57BL/6 mice, suggesting a strain dependent lethality (26). Outside of its canonical role in desmosomes and cell-to-cell adhesion, *Dsg2* has also been detected in embryonic stem cells lacking desmosomes, where it supports embryonic stem cell proliferation and embryo survival (26). Consistent with this, a related study from our laboratory also demonstrated the viability of a hypomorphic whole body *Dsg2* knock out in C57BL/6 mice (wherein low/residual levels of *Dsg2* are detectable) (9). This hypomorphic allele is not embryonic lethal but does manifest a pathological response, particularly an enlarged and fibrotic heart (9). DSG2 has also been reported to regulate actin assembly through interaction with integrin-β_8_ to promote angiogenesis in systemic sclerosis microvascular endothelial cells (27), and to serve a pro-survival role in intestinal epithelial tissue (28).

Further evidence to support DSG2 as a unique desmosomal protein with a biological role outside of desmosomes, is the selective expression of DSG2 by non-desmosome forming cells e.g., endothelial cells (ECs) (9, 29). Indeed, DSG2 is documented to assist in the survival, proliferation, migration, and angiogenesis of endothelial progenitor cells and some ECs (9). Interestingly, a study of microvascular ECs has shown that DSG2 interaction with integrin-β_8_ delivers pro-angiogenic signals via the Rho family of GTPases, focal adhesion kinase (FAK), Mothers against decapentaplegic homolog1/5(SMAD1/5), and extracellular signal-regulated kinase (ERK) 1/2 (27). More recently, we also documented that DSG2 is expressed by insulin-producing β-cells, with *in silico* analysis revealing that *DSG2* is in the top 10% of abundance of genes expressed by human islets (30).

**Figure 3:**
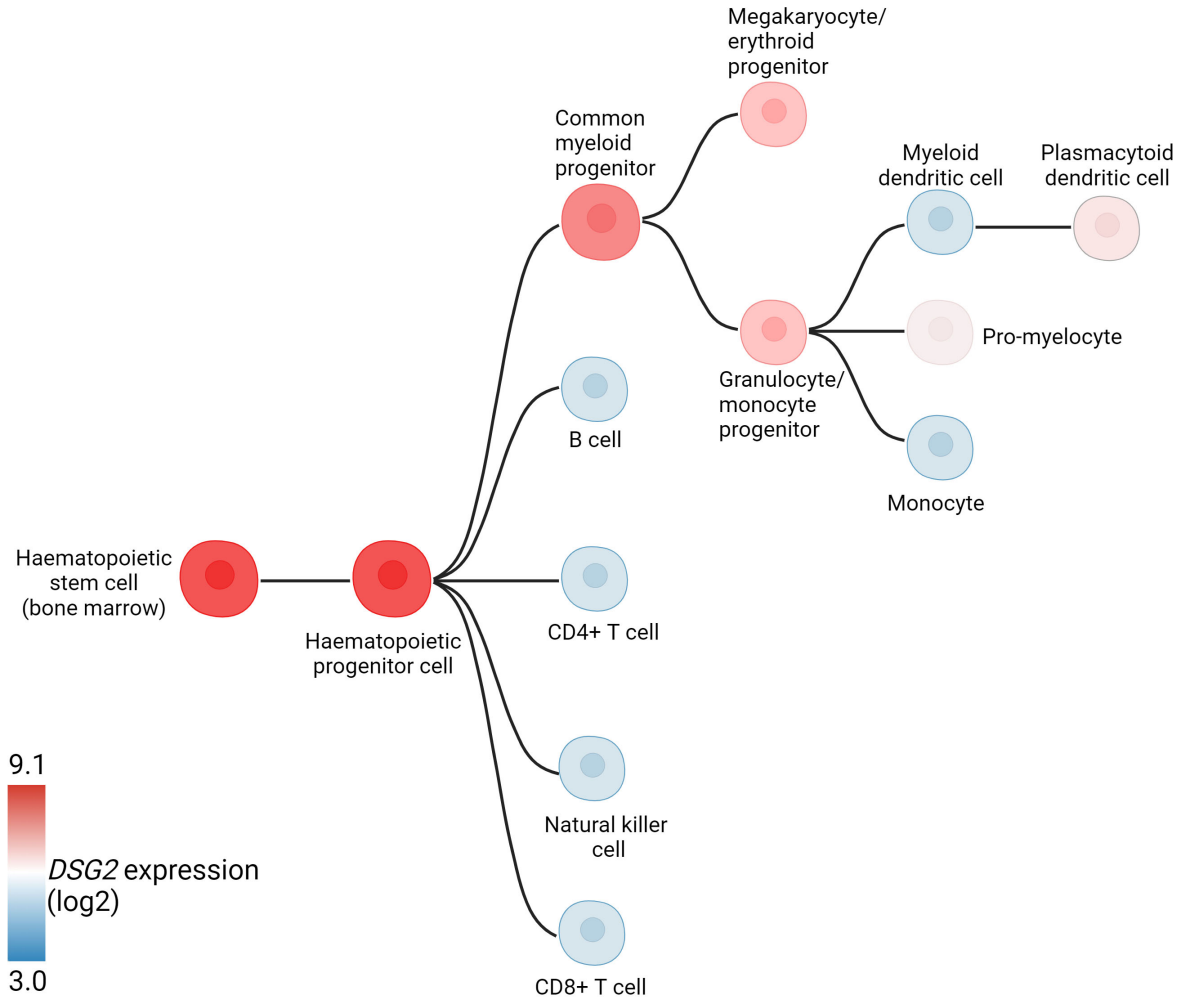
Hematopoietic hierarchical differentiation tree indicating *DSG2* expression (log2) at different stages of differentiation. Adapted from BloodSpot (31).

DSG2 has also been identified on non-desmosome forming haemopoietic progenitor cells. Surface expression of DSG2 has been identified on circulating CD34^+^CD45^dim^ hematopoietic progenitors (peripheral blood and cord blood), while other desmosomal components were noticeably absent (9). Interestingly, while DSG2 has been documented on progenitor and immature cells, its gene (**Figure 3**) and cell surface expression diminishes over the course of cellular differentiation (9, 31). The solitary expression of DSG2, and absence of other supporting desmosomal components, is uncharacteristic and suggests an alternative role for DSG2 within the hematological compartment. It is tempting to speculate that important similarities may emerge between DSG2-expressing stem cells and DSG2-expressing cancer cells. For example, the expression of DSG2 on pluripotent stem cells (PSC), is suggested to contribute to PSC self-renewal and pluripotency via regulation of β-catenin localization (32).

## 4 The role of DSG2 in cancer signaling and its prognostic value

### 4.1 DSG2 as a biomarker

**Figure 4:**
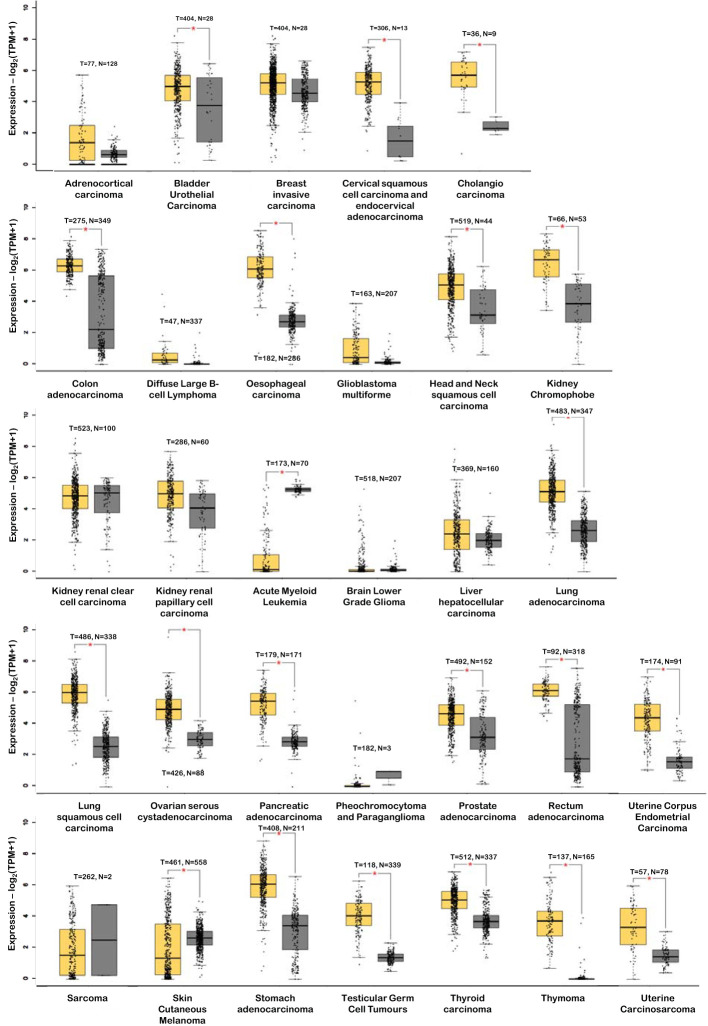
*DSG2* gene expression in tumor (T – in yellow) vs normal (N – in grey) tissue. *p<0.05. Data generated from GEPIA2 (33).

**Figure 5:**
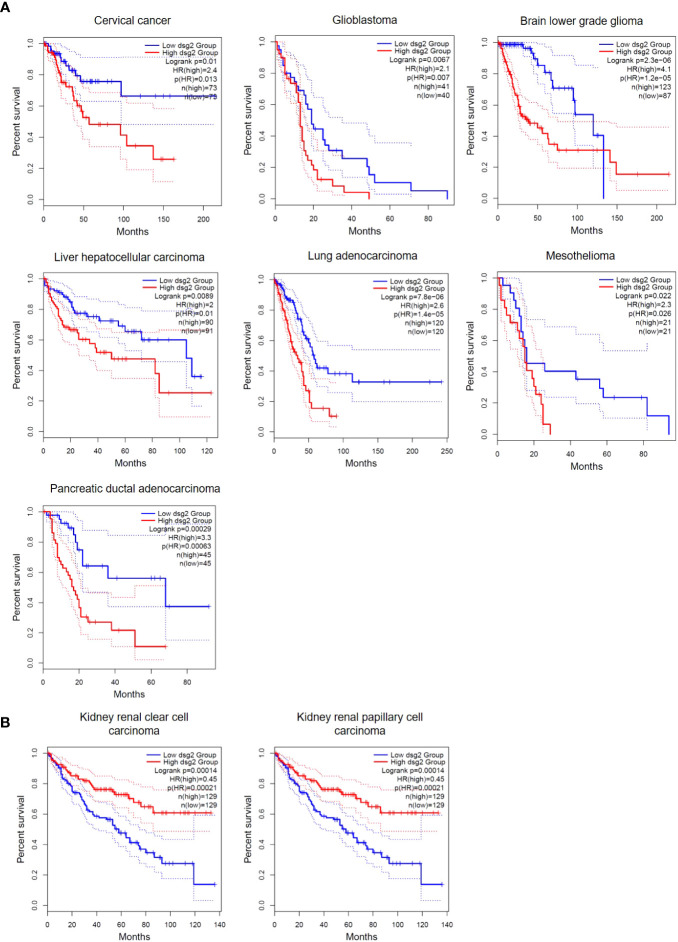
Overall Survival differences in *DSG2*-low vs *DSG2*-high expressing patients. **(A)** cervical cancer, glioblastoma, brain lower grade glioma, liver hepatocellular carcinoma, lung adenocarcinoma, mesothelioma and pancreatic ductal adenocarcinoma patients with significantly reduced Log rank OS in DSG2-high population, **(B)** kidney renal clear and papillary cell carcinoma patients with significantly increased Log rank OS in DSG2-high populations. Data generated from GEPIA2 (33).

Accumulating evidence suggests that *DSG2* is dysregulated in human cancers. This observation creates an opportunity for DSG2 to be a viable biomarker across a myriad of cancers, particularly as a predictive biomarker for disease progression. **Figure 4** shows analysis of publicly available *in silico* RNA seq datasets (Gene Expression Profiling Interactive Analysis, GEPIA2) demonstrating that *DSG2* gene expression is significantly upregulated in cancers such as lung squamous cell carcinoma, head and neck adenocarcinoma, pancreatic adenocarcinoma, prostate adenocarcinoma and stomach adenocarcinoma relative to normal healthy tissue (33). Overall survival analyses in GEPIA2 also indicate that high *DSG2* expression correlates with poor survival outcomes for patients with cancers including cervix, lung and pancreas (**Figure 5**) (33). In prognostic studies, high levels of *DSG2* resulted in worse clinical outcomes for patients with melanoma, multiple myeloma, basal cell carcinoma (BCC), squamous cell carcinoma (SCC), squamous cell lung carcinoma, ovarian cancer, head and neck SCC, and hepatocellular carcinoma (**Table 1**). In curious contrast, colon, gastric, prostate, and high-grade serous ovarian carcinoma patients presented with poor outcomes when *DSG2* levels were low (**Table 1**).

**Table 1:** DSG2 as a prognostic marker.

Cancer type	Role	Reference
**Basal cell carcinoma**	High DSG2 expression in BCC tumors compared to normal skin	(34–36)
**Bladder cancer**	Identified in a 11-gene prognostic signature (low *Dsg2*), with abnormality in >4 genes predicting poor prognosis	(37)
**Cervical cancer**	High *DSG2*/DSG2 expression predicative of poor clinical outcome	(38, 39)
	Identified in a gene prognostic signature (high *Dsg2)*, with a high-risk score in the genes predicting poor prognosis	(40–42)
	DSG2-AS1 lncRNA is expressed in EVs across all stages of disease and expression is significantly higher in cervical cancer patients compared to healthy controls, and is associated with poorer overall survival	(43)
**Colon cancer**	Low DSG2 expression associated with poor clinical outcome	(44)
**Endometrial cancer**	DSG2 identified to be a potential protein associated with endometrial cancer	(45)
**Extrahepatic cholangiocarcinoma**	No/low DSG2 expression associated with poor prognosis	(46)
**Gallbladder carcinoma**	High DSG2 expression associated with better overall survival, with loss promoting invasion and metastasis	(47)
**Gastric cancer**	Low DSG2 expression associated with poor clinical outcome	(48)
	Abnormal (non-membranous) localization of DSG2 in gastric cancer tissue	(49, 50)
	*DSG2* identified as predictive biomarker of the pathological response to neoadjuvant chemotherapy regimen	(51)
**Glioblastoma (GBM)**	High *DSG2* expression in GBM compared to normal brain tissue	(52)
**Hepatocellular carcinoma**	High DSG2 expression predictive of poor clinical outcome	(53)
**Laryngeal carcinoma**	High DSG2 expression is correlated with poorer survival and distant metastasis, high levels of DSG2 found in patient plasma	(54)
**Lung adenocarcinoma**	High *DSG2* expression positively associated with tumor size and lymph node metastasis and shorter overall survival	(55)
	Coxsackievirus/adenovirus receptors and DSG2 are highly co-expressed in early stages and associated with poorer overall survival	(56)
	Identified in a 10-gene signature, with high expression predicting poorer overall survival	(57)
**Melanoma**	High *DSG2* expression predictive of poor clinical outcome	(13)
**Multiple myeloma**	High *DSG2* expression predictive of poor clinical outcome	(58)
	Upregulation of *DSG2* in all 3 stages of the disease in the t(4;14) subgroup	(59)
**Esophagogastric junctional adenocarcinoma (EJA)**	Serum DSG2 was upregulated in EJA patients compared to healthy controls	(60)
**Ovarian cancer**	DSG2 is overexpressed and high serum levels of DSG2 lead to worse progression free survival	(61)
	High plasma levels of DSG2 identified in a signature of genes as a predictive indicator of poor prognosis	(62)
	DSG2 is overexpressed in novel subtype I compared to novel subtype II	(63)
	Low DSG2 expression associated with poor clinical outcome in high grade serous ovarian carcinoma	(64)
**Pancreatic adenocarcinoma (PDAC)**	DSG2 is expressed and upregulated in the serum of PDAC patients	(65)
	High DSG2 is predictive of poorer survival	(66)
	Identified in an acidosis-related gene signature used to predict poor prognosis and correlated with tumor immune infiltration	(67)
	Identified in a telomere 3-gene related prognostic signature, with higher DSG2 in PDAC tissue compared to normal pancreas	(68)
	Low DSG2 expression compared to normal tissue	(69)
**Prostate cancer**	Analysis of circulating tumor cells of pre-ADT (androgen deprivation therapy) samples found *DSG2* as a gene predictive of nonresponse	(70)
	Low DSG2 expression associated with poor clinical outcome	(71)
	Loss of DSG2 and E-cadherin was associated with poor prognosis, and a shorter biochemical recurrence-free survival. Observed a negative correlation between DSG2 expression, serum PSA, Gleason score and pathological stage	(72)
	Identified in an 8-gene signature (high Dsg2) that is associated with increased metastasis	(73)
**Squamous cell carcinoma (SCC)**	High DSG2 expression correlates with grade, type and risk of metastasis	(74)
	High DSG2 expression in SCC tumors compared to normal skin	(34, 75)
	High DSG2 expression predicative of poor clinical outcome	(76)
	High *DSG2* expression was associated with failure of treatment in early stages and recurrence of disease	(77)
	High *DSG2* expression identified in a signature of genes as a indicating poor prognosis	(78)
	Serum DSG2 was upregulated in esophageal SSC patients compared to healthy controls	(60)
	DSG2 protein expression cannot be used as a predictor of esophageal SCC patient outcome	(79)

Key: Red – high DSG2 expression correlates with poor outcome, blue – low DSG2 expression correlates with poor outcome, yellow – high DSG2 expression correlates with good outcome, grey – no preferable outcome.

### 4.2 DSG2 as a signal transducer

It is well established that the lateral mobility of cell surface proteins like DSG2 is critical for maintaining membrane dynamics (80–82). This highly complex and fluid membrane activity is vital for intracellular signaling through receptor activation as well as intercellular communication through vesicles, and recycling of membrane components. It has been reported by us and others that DSG2 promotes cancer cell migration, invasion, and proliferation in cancers such as SCC, breast, cervical, colon, and lung cancer (**Table 2**). More recently, we have also demonstrated that DSG2 facilitates cancer cell adhesion in multiple myeloma (9). Here we discuss the different signaling pathways and cellular functions that are regulated by DSG2 and the impact this has on cancer progression.

**Table 2:** DSG2 function in cancer.

Cancer type	Role of DSG2	Reference
**Basal cell carcinoma**	Promotes BCC development, enhances SHH signaling, activation of phosphorylated Stat, and regulation of Gli1	(83)
**Bladder cancer**	DSG2 expression is higher in cells also expressing PG	(84)
**Breast cancer**	DSG2 and E-cadherin suppress invasion and motility *in vitro*	(85)
	Interplay between DSG2 and hypoxia controls metastasis: DSG2 expression promotes metastatic colonization and tumor growth.	(86)
	DSG2 is a substrate of matriptase	(87)
	DSG2 is a receptor for eFABP4 that stimulates breast cancer growth through an ERK–NRF2 signaling axis	(68)
	The shed extracellular domain of DSG2 is expressed in breast tumor samples	(88)
**Cancer stem cells**	Autocrine loop established between DSG2 and Wnt/Beta-catenin signaling pathway	(89)
	DSG2 interacts with CD133 in stem cells from ovarian clear cell carcinoma, and CD133 knockdown reduces DSG2 expression	(90)
**Cervical cancer**	Promotes cell proliferation, migration, and invasion via mediating MAPK pathway	(12)
	Promotes cell proliferation and migration	(38)
**Choriocarcinoma**	DSG2 directly interacts with PLAC1, with mutations in the ZP-N domain of PLAC1 completely disrupting interactions between PLAC1 and DSG2	(91)
**Colon cancer**	Promotes tumor growth, and enhances EGFR signaling	(92)
	Pinin activates EGFR/ERK signaling pathways through the upregulation of DSG2 to promote proliferation, invasion, and metastasis	(93)
	DSG2 has a role in the tumor aggregation process with E-cadherin, with loss reducing cell aggregation	(94)
**Gallbladder carcinoma**	Inhibits cell proliferation, migration, invasion, and tumor growth via Src signaling	(95)
	DSG2 knockdown led to promotion of cell invasion and metastasis	(47)
**Gastric cancer**	TROP2 downregulates DSG2 to promote invasion and migration through DSG2/PG/β-Catenin pathways	(96)
**Lung adenocarcinoma**	Promotes cell proliferation, migration, and increases resistance to the EGFR tyrosine kinase inhibitor Osimertinib, via the EGFR-Src-Rac1-PAK1 signaling pathway.	(16)
**Melanoma**	Promotes vasculogenic mimicry	(13)
	Attenuates migration which is mediated by downregulation of secretogranin II	(97)
**Multiple Myeloma**	Promotes cell adhesion	(58)
	Multiple myeloma SET (MMSET) (with isoforms upregulated in t(4;14)) suppression reduces expression DSG2	(98)
**Non-small cell lung cancer**	Promotes NSCLC cell proliferation and xenograft development	(11)
**Ovarian cancer**	Slug suppresses the expression of demosomal junction component DSG2, reducing intercellular adhesion	(99)
**Pancreatic adenocarcinoma**	Inhibits cell migration, invasion, and regulates PG in an ERK dependent manner	(100)
	DSG2 is a substrate of kallikrein-7	(69)
	DSG2 expression is associated with an immunosuppressive TME (negative correlation with CD8 T cells, follicular helper T cells and eosinophil infiltration)	(101)
**Squamous cell carcinoma (SCC)**	Reduced DSG2 levels correlated with decreased strength of cell-cell adhesion	(102)
	EGFR inhibition increases DSG2 expression and desmosome formation	(103)
	Maintains cell adhesion, and is associated with lipid rafts (Cav-1)	(104)
	Promotes cell proliferation, regulates SHH signaling, and accelerates squamous-derived tumorigenesis	(105)
	Enhances EGFR activation via Src and Cav-1, and promotes cell proliferation and migration	(15)
	Modulates EV release from SCC keratinocytes	(106)
	Promotes SCC tumor development, downregulates miR-146a, resulting in increased production of IL-8, and is packaged and released in small EVs	(14)
	DSG2 interacts with miR-146a and IL-8 to drive immune checkpoint inhibitor resistance	(107)
	An inverse molecular switch from DSG3 to DSG2 during oral SCC tumor progression	(108)
	Snail enhanced the degradation of E-cadherin and DSG2	(109)
**Thyroid cancer**	Inhibits migration and invasion via HGFR, c-Met/Src/Rac1 signaling axis	(110)

Key: Red - pro-tumorigenic, blue - anti-tumorigenic, grey- DSG2 as an interacting protein in cancer.

#### 4.2.1 Epidermal Growth Factor Receptor

DSG2 has been previously reported to facilitate EGFR activity; DSG2/EGFR interaction has demonstrated importance in the development of SCC (15), colon cancer (92), and lung adenocarcinoma (16). In response to EGF ligand stimulation, DSG2 is known to be involved in the activation of EGFR, c-Src and Signal transducer and activator of transcription (STAT3), which supports cancer cell growth and migration (15). In SCC, DSG2 co-localizes with the EGFR and promotes the activation of c-Src mediated signaling by displacing EGFR from the inhibitory lipid raft microenvironment, thereby making it available for ligand binding and signaling (104, 111). Overexpression of DSG2 in human SCC cells is also linked to enhanced cancer cell proliferation and migration (also via activation of c-Src) (15). The EGF/EGFR axis directly initiates the phosphorylation of membrane resident catenins that go on to facilitate links between junctional proteins in desmosomes and the actin cytoskeleton or intermediate filaments (112). This impacts cell migration with DSG2/EGF/EGFR-dependent tyrosine phosphorylation of plakoglobin being able to shift the cell from an ‘adhesive’ to a ‘migratory’ phenotype for increased epithelial cell motility (112). The EGFR is also reported to stabilize DSG2 at cellular junctions in an A disintegrin and metalloprotease (ADAM)-dependent manner (113). A study on colon cancer also showed that phosphorylation of the EGFR and downstream signal activation is enhanced by DSG2, where downregulation of DSG2 decreases EGF-induced cell proliferation and suppresses *in vivo* xenograft tumor growth (92). Additionally, in lung adenocarcinoma, loss of DSG2 (via gene silencing) leads to the nuclear translocation of EGFR, as well as suppression of EGFR signaling via the Src-Rac1-PAK1 pathway (16). Thus, through its role as a regulator of a key protein such as EGFR, DSG2 has far-reaching signaling effects that can potentiate oncogenic outcomes.

#### 4.2.2 Other signaling pathways

Additional pathways that have been linked to DSG2 include the mitogen-activated protein kinase (MAPK) and the sonic hedgehog (SHH) in cervical cancer and BCC respectively. In cervical cancer, DSG2 was observed to promote cell proliferation, migration, and invasion by modulating phospho Mitogen-activated protein kinase kinase (pMEK) and phospho Extracellular signal-regulated kinase (ERK) within the MAPK pathway (12). Whilst in BCC, DSG2 was shown to enhance downstream SHH signaling through the secretion of cytokines, upregulation of receptors (e.g., urokinase plasminogen activator surface receptor (uPAR) and interleukin (IL)-6R) and exosome secretion, leading to STAT3 phosphorylation and activation, and potentiating GLI1 expression (83). DSG2 also has reported roles in the progression of non-small cell lung cancer tumors *in vitro* and *in vivo*, with reduced expression of DSG2 causing cell cycle arrest at the G1 phase (11). DSG2 has also been reported to be regulated by Hypoxia-inducible factor 1 (HIF1)-α to support metastasis in breast cancer (85).

While the aforementioned pathways converge to promote tumor growth, there are also reports illustrating that reduced DSG2 levels promote tumorigenesis. For example, in thyroid cancer, DSG2 depletion led to increased cell migration and metastasis via activation of the hepatocyte growth factor receptor (HGFR, c-Met)/Src/Rac1 signaling pathway (110). In gallbladder carcinoma, loss of DSG2 was associated with cancer progression and resistance to EGFR-targeted therapy through activation of Src kinase (95). Furthermore, a study in pancreatic adenocarcinoma suggested that loss of DSG2 promoted cell migration and invasion by regulating the desmosomal linker protein plakoglobin (PG) in an ERK-dependent manner (100). Finally, in gastric cancer, DSG2 was reportedly down-regulated by cell-surface glycoprotein TROP2 to promote invasion and migration through DSG2/PG/β-catenin pathways (96).

#### 4.2.3 Extracellular vesicle modulation

Intercellular communication, which is essential for all biological processes, is accomplished by various means, including cell-cell and cell-microenvironment signaling, the release of modulating soluble molecules such as cytokines, chemokines, and growth factors, and the secretion of extracellular vesicles (EVs, cargo-carrying messengers) (114). Subversion of these normal signaling events disrupts normal tissue homeostasis and morphogenesis and underlies pathogenic signaling during cancer progression. EVs are lipid bilayer-bounded organelles with unique membrane-associated proteins and lipids carrying a cargo of proteins, lipids, and/or genetic material (115). The three major subpopulations of EVs include apoptotic bodies, microvesicles, and exosomes, distinguished by their mechanism of biogenesis, size, content, and biological functions. Most importantly, EVs can serve as important biomarkers for disease diagnosis and prognosis in the clinical setting (116, 117).

Exosomes are generated by membrane invagination into the early endosomal compartment and, upon a second round of membrane invagination, mature into multivesicular bodies (MVB), or late endosomes. MVB are degraded following fusion with lysosomes or trafficked back to the plasma membrane for release as encapsulated exosomes. The presence of DSG2 in exosomes was first reported in 2017 (106). A few years later, DSG2 was documented to modulate exosome biogenesis in a palmitoylation-dependent manner, suggesting lipid modification, membrane localization, and protein-protein interaction are critical determinants (14). Furthermore, DSG2 has been shown to regulate proteins, such as flotillin and caveolin, involved in the early steps of endosomal processing (14, 106).

It has been reported that DSG2 plays a role in modulating membrane dynamics through lipid rafts and the endosomal pathway leading to exosome generation and release. Caveolin-1, a known interactor of DSG2, initiates membrane invagination into vesicles of approximately 60-80 nm in diameter and, similar to exosomes, caveolae and lipid rafts are enriched in cholesterols and sphingolipids (118). Additionally, DSG2 undergoes ectodomain shedding mediated by ADAM10 and/or ADAM17, resulting in a 95-kDa ectodomain cleaved product and a 65-kDa membrane-spanning fragment (106, 119). This 65-kDa C-terminal fragment is detected in lipid rafts (104) as well as exosomes (106). Interestingly, DSG2 co-localizes with Sec3, a protein component of the exocyst, an octameric protein complex that facilitates the transport of secretory organelles from the Golgi to the plasma membrane (120). At the plasma membrane, these exocyst organelles dock at desmosomes prior to vesicle fusion. Exocysts do not fuse with the endosomes (121) and are distinct from multivesicular bodies, thus, inhibitors of the endocytic pathways do not affect exocyst biogenesis. These studies highlight many novel critical roles for DSG2 in intracellular signaling and vesicle biogenesis from initiating endosome formation to vesicle docking.

The mechanisms by which exosomes serve as signaling mediators is cargo dependent (122). The list of components detected in exosomes is ever expanding to include lipids, proteins, DNA, and various species of RNA (mRNA, miRNA, lncRNA, and cRNA) (123). DSG2 alters the miRNA levels and cytokine/chemokine profiles not only in cells but also in exosomes (14, 124). The direct impact of these factors modulated by DSG2 requires further study, but early studies show that exosomes derived from DSG2 overexpressing cancer cells enhance dermal fibroblast cell growth, demonstrating that DSG2 expression can modulate the tumor microenvironment. Most importantly, we can demonstrate that intercellular communication through cell-cell adhesion, cytokine release, and secretion of modified EVs are well-coordinated by DSG2. For instance, studies in SCC have also shown that DSG2 is involved in the release of EVs. Briefly, head and neck SCC EVs are enriched for DSG2 and overexpression of DSG2 is shown to increase the release of EVs which can enhance fibroblast cell growth (107). In line with this, EVs released from SCCs overexpressing DSG2 promote SCC tumor growth *in vivo* and contain cargo that is pro-tumorigenic, e.g. IL-8 and IL-6 (14).

### 4.3 Vasculogenic mimicry

The vasculature plays a pivotal role in tumor growth and metastasis. Tumors can develop a blood supply not only by promoting angiogenesis, where blood vessels are formed by ECs, but also by vessel-like structures directly formed by cancer cells, in a process known as vasculogenic mimicry (VM) (125–127). Abundant VM has been linked to increased invasion, high tumor grade, metastasis, and overall poor survival for several cancer types (128). Of relevance here, DSG2 is an important regulator of both angiogenesis and VM; for example, in a mouse model of melanoma, loss of *Dsg2* expression in the host was associated with fewer EC-lined tumor vessels (9). Furthermore, elevated DSG2 on melanoma cells promoted the formation of VM structures *in vitro*; in support of a pro-vascular phenotype, melanoma cancer cells expressing high levels of *DSG2* also overexpress VM-associated genes (13). While further studies are required to fully elucidate the role of DSG2 in VM formation, data to-date suggest that targeting DSG2 in melanoma may inhibit VM formation and suppress tumor growth and metastasis.

### 4.4 The varied functions of DSG2 across different cancer types

**Figure 6:**
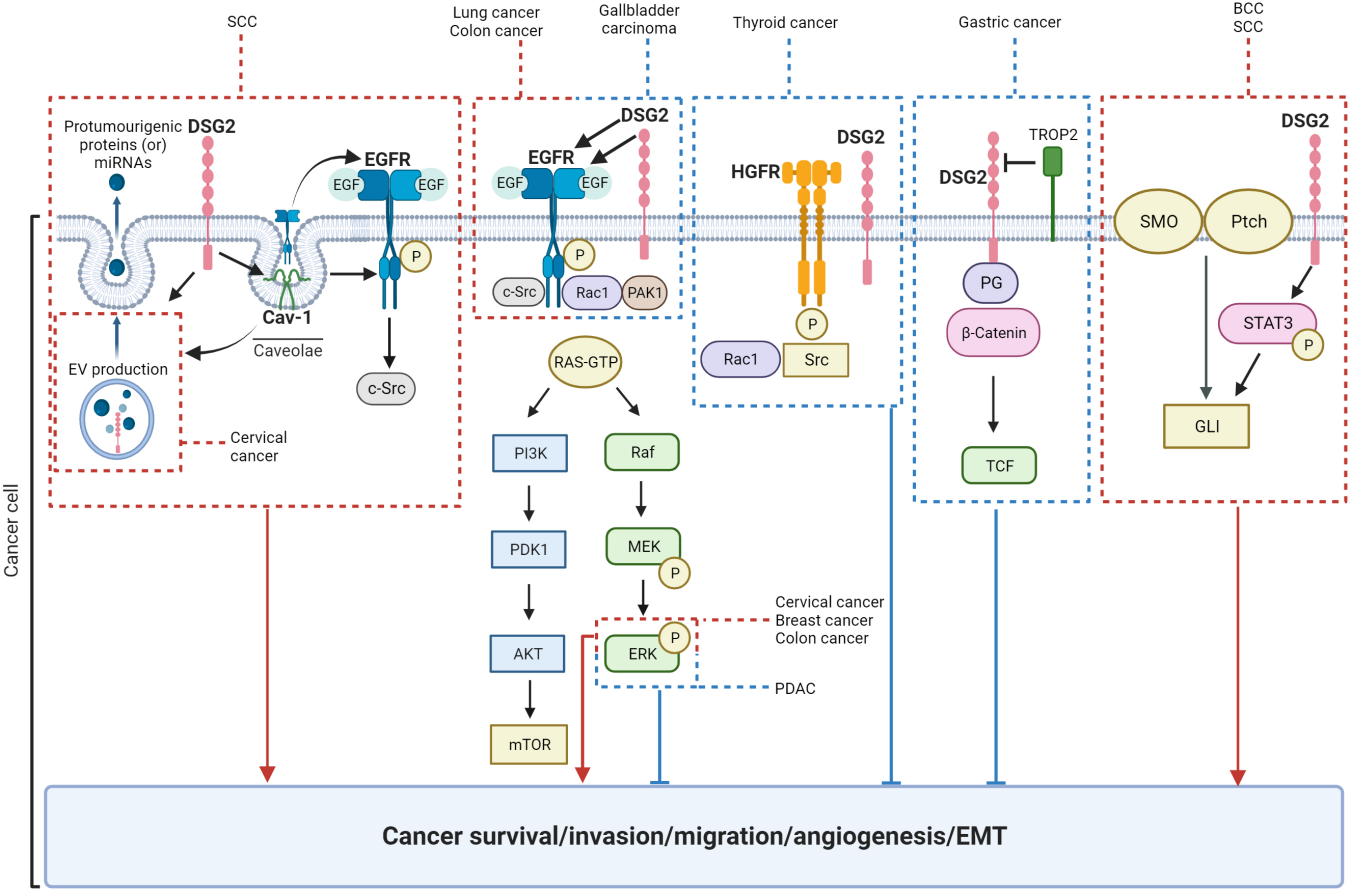
Schematic of signaling pathways known to be regulated by DSG2. Key: red = pro-tumorigenic, blue = anti-tumorigenic.

Current literature suggests that DSG2 plays a broad and significant role in regulating various cancer cell functions, including cell proliferation, survival, migration, invasion, adhesion, cell cycle progression, vesicle secretion, and tumor development (**Table 2**; **Figure 6**). Notably, conflicting roles for DSG2 in cancer progression have emerged and at present appear to coincide with the different cancer types. To the best of our knowledge, **Table 2** summarizes the current landscape for DSG2 in cancer and suggests that for the majority of cancers, elevated expression levels of DSG2 promotes signaling pathways that support cancer progression. Briefly, studies related to SCC, BCC, breast cancer, cervical cancer, colon cancer, lung cancer, multiple myeloma, and ovarian cancer are predominantly associated with a pro-tumorigenic function for DSG2. In contrast, reports implicating an anti-tumorigenic function for DSG2 exist for cancers of the gallbladder, thyroid, and the stomach. One potential explanation for these observed differences may involve the interplay between DSG2 and other cell surface expressed proteins associated with cell adhesion. For example, inflammation-induced reduction of DSG2 is linked to alterations in the expression of tight junction proteins, such as Claudin2 (129). Disruption of Claudins facilitates the infiltration of growth factors into the mucosa which are known to promote neoplastic transformation (130). This is supported by Wang et al, who documented that loss of DSG2 on gallbladder cancer cells promoted EMT with reduced expression of E-cadherin and increased expression of Snail (47).

We speculate that DSG2 may exist as both a vital part of desmosomes facilitating cell-to-cell adhesion as part of its canonical function, as well as an independent signal transducer. This leads to the inference that the mechanisms through which DSG2 influences cancer progression are influenced by the tumor microenvironment and the origin of the cancer itself. DSG2 has been shown to activate a variety of signaling pathways and proteins including MAPK, STAT3, growth factor receptors. To this end, DSG2-mediated EGFR signaling consistently promotes cancer progression whereas DSG2-mediated HGFR signaling suggests an inhibition of cancer growth (**Figure 6**). Taking the heterogeneity within individual cancers into consideration, it is evident that the role DSG2 plays in tumorigenesis is complex and context dependent. Therefore, the potential for DSG2 to act as a prognostic biomarker is context dependent and influenced by factors such as organ/tissue type, cancer cell location within a tissue, genetic mutations, as well as the broader tumor microenvironment. To date, there is no distinguishing feature that identifies the ‘tumor promoting’ from the ‘tumor suppressing’ role of DSG2 in cancer. To gain clarification, studies that stratify tumor stage, subtype, and genetic drivers based on DSG2 levels across multiple cancers would be informative.

## 5 Cleavage of DSG2

Like other transmembrane junctional proteins, DSG2 can be cleaved by various proteases. DSG2 is predicted to have several cleavage sites; it has been reported that full length DSG2 (150 kDa) can be cleaved into two or three cleaved fragments, i.e. a 110 kDa ectodomain (i.e., extracellular portion) fragment, a 95 kDa ectodomain fragment and a 65 kDa membrane-spanning fragment (104, 119, 131). The ectodomain of DSG2 can be cleaved by proteases including matrix metalloproteinase (MMP) 9 (103, 119), ADAM10 and ADAM17 (103, 113, 119, 132, 133), kallikrein 7 (69), matriptase (134), and γ-secretase (135). Interestingly, the intracellular portion of DSG2 can also be cleaved by caspase 3 and 8 (28, 136, 137) as well as calpain (28). Several studies have also shown that inflammatory mediators such as IL-1β, tissue necrosis factor (TNF)α, interferon (IFN)-γ as well as EGF can induce/increase both the extracellular and intracellular cleavage of DSG2 via activation of the aforementioned proteases (28, 103, 119, 132, 133, 136).

Within cancer, the ectodomain cleavage of DSG2 has been associated with decreased cell-cell adhesion (134) and decreased cell proliferation (119, 136). Yulis et al. (136) and Nava et al. (28) showed that IFNγ/TNFα-induced cleavage of DSG2 caused cancer cell apoptosis. Reports, including our own study, indicate that the cleaved ectodomain of DSG2 can be detected in cell culture supernatant and serum (human and mouse) by ELISA (54, 61, 62, 65, 88, 104, 138). Liu et al. (60) demonstrated that the levels of serum DSG2 are significantly higher in patients with esophageal squamous cell carcinoma and esophagogastric junction adenocarcinoma. Kim et al. (61). reported that patients with ovarian cancer who had high circulating levels of soluble DSG2 had poorer progression-free survival compared to healthy controls (i.e., a median survival of 16 months vs. 26 months). Notably, while proteases that cleave DSG2 have been identified, little is known about how the circulating DSG2 fragments cause progression of cancer, and addressing this requires further research.

## 6 DSG2 and viruses

Also of interest is the ability of DSG2 to act as a high-affinity receptor for some species B human adenoviruses (e.g. HAd3, 7, 11 and 14), with the second and third ectodomains (EC2 and EC3) of DSG2 binding to the fiber knob protein of the virus (139). When bound to DSG2, the adenovirus activates the MAPK pathway to upregulate the proteases ADAM17 and MMP9 that then cleave the extracellular domain of DSG2 releasing it from the cell surface (140). The significance of this process is yet to be determined, but it is evident that adenovirus binding to DSG2 leads to the transient opening of intercellular junctions. Recent reports have shown that Ad3 fiber knob-containing recombinant junction opening proteins (JO-1,2,3,4) are able to bind to and trigger DSG2 shedding, causing a junctional opening (141). Relevant to this review, when administered *in vivo* in combination with chemotherapy, JO1/4 increased the efficacy of chemotherapeutic drugs, resulting in decreased tumor growth and delayed drug resistance (142–144). Capitalizing on this biological function for better cancer outcomes is yet to be fully realized and warrants careful consideration.

Furthermore, evidence supports a correlation between DSG2 and human papillomavirus (HPV) linked cancers. Zhao et al. showed that *DSG2* expression levels were related to HPV status, with all HPV-positive cervical cancer patients exhibiting high *DSG2* levels (40). This is supported by a recent study wherein DSG2 levels were elevated in HPV-positive head and neck SCCs when compared to HPV-negative tumors (107). Moreover, DSG2 expression was four times higher in HPV-positive patients who failed to respond to immune checkpoint inhibitors (ICI) compared to those who did respond (107). These results suggest that DSG2 may play an underappreciated role in HPV-induced cervical cancer.

## 7 Future perspectives

From a potential therapeutic standpoint, since there is evidence to suggest that blocking DSG2 could achieve tumor destruction (**Table 2**), there may be therapeutic benefit in utilizing DSG2 as either as a monotherapy (e.g., monoclonal antibody, small molecule inhibitors etc.) or in combination with chemotherapy, radiation, ICI, or other targeted therapies for select cancers. For instance, given that DSG2 promotes EGFR expression and EGF-mediated signaling in cancer cells, inhibition of DSG2 in combination with EGFR targeted therapy (e.g., Erlotinib) could be of clinical benefit. There is also building evidence suggesting that DSG2 plays a role in modulating ICI efficacy. More specifically, a study in head and neck SCC suggested that DSG2-mediated signaling negatively impacted ICI responses (107). Based on these observations, targeting DSG2, prior to (or in combination) with ICI treatment, may improve clinical outcomes for current ICI non-responders. Alternatively, given that DSG2 is a functional receptor for select adenoviruses, the possibility exists for its use to improve the systemic delivery of oncolytic viruses (145). The use of JO proteins that target DSG2 and cause junctional opening is yet another promising therapeutic option that could be considered in combination with chemotherapeutics (144). Notably, given the current consensus that DSG2 has varied roles in cancer progression (as described in **Table 2**), careful consideration is warranted.

## 8 Conclusions

In summary, this review presents evidence that DSG2 is unlike other desmosomal components in its capacity to act as a solitary protein with a broader repertoire of biological roles. This is of clinical interest as DSG2 is increasingly implicated in the poor prognosis of patients with cancer. To capitalize on DSG2 as a potential biomarker for cancer prognosis and treatment, we continue to acquire knowledge of its association with (and consequences of) key intracellular signaling pathways. The physical positioning of DSG2 within the plasma membrane of cells provides ample opportunity for it to directly engage with various factors in the local microenvironment (e.g. growth factors and integrins) that initiate cell signaling pathways. These non-canonical roles for DSG2 are undoubtedly advantageous for cancerous cells who thrive on improved cell survival, proliferation and migration. Intersecting these cellular advantages via targeting DSG2 in cancer could provide clinical benefit.

## Author contributions

KM: Conceptualization, Writing – original draft, Writing – review & editing. CF: Conceptualization, Writing – original draft, Writing – review & editing. BM: Writing – original draft, Writing – review & editing. MO: Writing – original draft. ED: Writing – review & editing. MS: Writing – review & editing. LE: Writing – review & editing. MM: Writing – original draft, Writing – review & editing. CB: Writing – original draft, Writing – review & editing.
